# Prevalence of Neutralising Antibodies to HCoV-NL63 in Healthy Adults in Australia

**DOI:** 10.3390/v13081618

**Published:** 2021-08-16

**Authors:** Sean A. Lynch, Kanta Subbarao, Siddhartha Mahanty, Bridget E. Barber, Eileen V. Roulis, Lia van der Hoek, James S. McCarthy, Kirsten M. Spann

**Affiliations:** 1 QIMR Berghofer Medical Research Institute, Herston, QLD 4006, Australia; Sean.Lynch@qimrberghofer.edu.au (S.A.L.); Bridget.Barber@qimrberghofer.edu.au (B.E.B.); James.mccarthy@unimelb.edu.au (J.S.M.); 2 WHO Collaborating Centre for Reference and Research on Influenza, Department of Microbiology and Immunology, Melbourne, VIC 3000, Australia; kanta.subbarao@influenzacentre.org; 3 The Peter Doherty Institute for Infection and Immunity, University of Melbourne, Melbourne, VIC 3000, Australia; siddhartha.mahanty@unimelb.edu.au; 4 Australian Red Cross Lifeblood, Kelvin Grove, QLD 4059, Australia; ERoulis@redcrossblood.org.au; 5 Laboratory of Experimental Virology, Department of Medical Microbiology and Infection Prevention, Amsterdam Institute for Infection & Immunity, Amsterdam UMC, University of Amsterdam, 1105 AZ Amsterdam, The Netherlands; c.m.vanderhoek@amsterdamumc.nl; 6 Victorian Infectious Diseases Service, The Royal Melbourne Hospital, Parkville, VIC 3050, Australia; 7 Centre for Immunology and Infection Control, School of Biomedical Science, Faculty of Health, Queensland University of Technology, South Brisbane, QLD 4007, Australia

**Keywords:** HCoV-NL63, neutralising antibody titre, healthy adults

## Abstract

The COVID-19 pandemic has highlighted the importance of understanding the immune response to seasonal human coronavirus (HCoV) infections such as HCoV-NL63, how existing neutralising antibodies to HCoV may modulate responses to SARS-CoV-2 infection, and the utility of seasonal HCoV as human challenge models. Therefore, in this study we quantified HCoV-NL63 neutralising antibody titres in a healthy adult population using plasma from 100 blood donors in Australia. A microneutralisation assay was performed with plasma diluted from 1:10 to 1:160 and tested with the HCoV-NL63 Amsterdam-1 strain. Neutralising antibodies were detected in 71% of the plasma samples, with a median geometric mean titre of 14. This titre was similar to those reported in convalescent sera taken from individuals 3–7 months following asymptomatic SARS-CoV-2 infection, and 2–3 years post-infection from symptomatic SARS-CoV-1 patients. HCoV-NL63 neutralising antibody titres decreased with increasing age (R^2^ = 0.042, *p* = 0.038), but did not differ by sex. Overall, this study demonstrates that neutralising antibody to HCoV-NL63 is detectable in approximately 71% of the healthy adult population of Australia. Similar titres did not impede the use of another seasonal human coronavirus (HCoV-229E) in a human challenge model, thus, HCoV-NL63 may be useful as a human challenge model for more pathogenic coronaviruses.

## 1. Introduction

Coronaviruses are positive-sense, single-stranded RNA viruses that infect a range of animals including humans. The current pandemic is caused by a betacoronavirus called SARS-CoV-2. Although this virus is believed to have spread to humans through zoonotic transmission, its wide dissemination has sparked renewed interest in the four globally endemic seasonal circulating human coronaviruses (HCoVs): HCoV-NL63, HCoV-OC43, HCoV-229E, and HCoV-HKU1. These viruses most commonly cause respiratory tract infections in children, with manifestations ranging from asymptomatic infection to croup, bronchiolitis, and pneumonia. However, they usually present as mild upper respiratory tract symptoms [1]. Within the *Coronaviridae* family, HCoV-NL63 is classified as an alphacoronavirus and, like SARS-CoV-2, it binds to angiotensin-converting enzyme 2 (ACE2) on the cell surface to mediate cellular entry [2].

HCoV-NL63 was first isolated in 2003 from the nasopharyngeal aspirate of a 7-month-old child with bronchiolitis, fever, and conjunctivitis in the Netherlands [3]. Although HCoV-NL63 has been associated with lower respiratory tract disease, infection is often asymptomatic, and therefore the true prevalence of infection is difficult to ascertain. In a study undertaken in Germany from November 1999 to October 2001, 1756 respiratory samples collected from children under 3 years who were hospitalised or visited outpatient clinics were tested for HCoV-NL63 by RT-PCR. The annual incidence of HCoV-NL63 infections was calculated as 7 per 1000 children, with a hospitalisation rate of 22 in 100,000 children [4]. A marked winter to spring seasonality in HCoV-NL63 infection has been reported in Western Europe and the United Kingdom, with the virus almost never detected in the summer [4,5].

Due to a lack of global surveillance for HCoV-NL63, its predominantly asymptomatic presentation, and low clinical impact in healthy adults, the seroprevalence of HCoV-NL63 in adults has not been defined. Clinical microbiology and seroprevalence studies of HCoV-NL63 have generally focused on children, and most recent studies have used assays against viral peptides rather than neutralization assays. In one study in the Netherlands, 75% of children between the ages of 2.5 and 3.5 years were seropositive for HCoV-NL63 [6]. However, immune protection against seasonal coronaviruses is short-lived. A longitudinal cohort study in Amsterdam in which serological assays were conducted in 10 healthy adult males 3–6 monthly for 35 years, showed that reinfection with seasonal coronaviruses was possible within 6 to 105 months after initial infection, and re-infections were most often observed after 12 months [7]. HCoV-NL63 infections in this study were, however, relatively rare, with an average of only 2.5 infections per individual detected over an average monitoring time of 20 years.

Interest in HCoVs has been renewed since the emergence of SARS-CoV-2, particularly in the effect of prior exposure to HCoVs on the outcome of subsequent SARS-CoV-2 infection [8], mediated by cross-reactive humoral or cellular immunity between SARS-CoV-2 and seasonal coronaviruses [9]. Cross-reactive immune responses to HCoVs may complicate the interpretation of serological studies for SARS-CoV-2, and may protect against [10,11] or enhance [12] the severity of COVID-19 disease. Recent interest in HCoVs has also focused on their potential as human challenge surrogates for SARS-CoV-2. They may be appropriate low virulence model viruses for human challenge studies to investigate the efficacy of antiviral treatments for COVID-19, and in vitro studies regarding cellular entry and responses to infection. In this context, HCoV-NL63 would be the most advantageous virus to use, as HCoV-HKU1 is difficult to propagate in cell lines, and the commercially available strains of both HCoV-OC43 and HCoV-229E are laboratory adapted high passage viruses that may not induce clinically relevant disease manifestations in humans [13]. However, if the population prevalence of high titre neutralising antibodies to HCoV-NL63 from which volunteers may be drawn is high, the feasibility of a challenge model may be reduced. We therefore conducted a cross-sectional study to determine the seroprevalence of neutralising antibodies against HCoV-NL63 in healthy adults in Australia.

## 2. Materials and Methods

### 2.1. Study Population

One hundred plasma samples from healthy blood donors were randomly selected from donated, SARS-CoV-2-negative blood products collected by Australian Red Cross Lifeblood in August, 2020 (ethics number 04092020). The mean age of donors was 48.3 years (range 19–74 years) and 56% were male. There was no difference between the median age of males and females. Donors were from five states in Australia; 67% from Queensland, 10% from New South Wales, 20% from the Northern Territory, 2% from South Australia, and 1% from Tasmania. More detailed demographic information, such as ethnicity of donors, was not available due to ethical and privacy constraints.

### 2.2. Virus Propagation and Titration

HCoV-NL63 (Amsterdam-1 strain) was used to infect LLC-MK2 (ATCC, CCL-7) cells at a multiplicity of infection of 0.1 in OptiMEM/2% fetal calf serum/1% antibiotic–antimycotic (Thermo Fisher Scientific, Scoresby, VIC, Australia) supplemented with 1 µg/mL tosyl phenylalanyl chloromethyl ketone (TPCK)-treated trypsin (Worthington Biochemical, Lakewood, NJ, USA). Cell culture supernatant and attached cells were collected after 4 days of incubation at 34 °C/5% CO_2_, when a cytopathic effect was visible and 75% of cells were detached. Supernatant was clarified at 1500× *g* for 5 min at 4 °C. The cell pellet was freeze/thawed three times to recover cell-associated virus and pooled with the clarified supernatant. The titre of stock virus was quantified by recording cytopathic effect caused by 10-fold dilutions in LLC-MK2 cells maintained in OptiMEM/1 µg/mL TPCK-treated trypsin. Virus titre was calculated using the Spearman–Karber algorithm.

### 2.3. Microneutralisation Assay

Plasma samples were heat-inactivated at 56 °C for 30 min and diluted 1:10 in OptiMEM/GlutaMAX (ThermoFisher). A log2 dilution series was prepared with 1:160 as the final dilution. Each dilution of plasma was incubated with 100 TCID_50_ HCoV-NL63/OptiMEM/0.5% BSA for 1 h at room temperature. Aliquots of the plasma/virus mix were transferred to four replicate wells of confluent LLC-MK2 cells in 96-well plates. Control wells included on each plate in quadruplicate were no virus, no serum negative controls, and serum-free virus-exposed positive controls. Plates were incubated at 34 °C and 5% CO_2_, for 6 days, at which time they were fixed and stained with 20% methanol/0.1% crystal violet/ddH_2_O. The neutralisation titre was calculated using the Reed–Muench algorithm to identify the highest dilution of plasma that completely inhibited the cytopathic effect in 50% of replicate wells. The geometric mean titre (GMT) of two independent neutralisation assays for each plasma sample was calculated, using only those neutralisation titres for each sample that were within a 2-fold difference. Assays were repeated if neutralisation titres were more than 2-fold different in independent assays [14,15].

### 2.4. Statistical Analyses

The Kruskal–Wallis test was used to compare titres between age groups, and the association between age and GMT was determined by linear regression of log_10_ transformed GMT. The Mann–Whitney *U* test was used to compare GMTs between males and females, and Fisher’s exact test was used to compare the prevalence of neutralising antibodies.

## 3. Results

**Figure 1:**
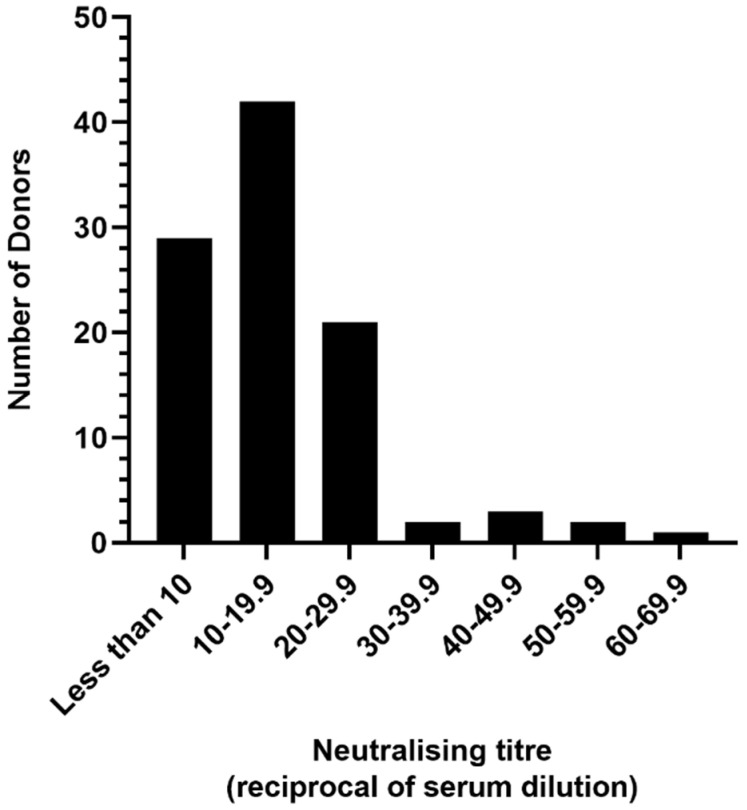
Geometric mean HCoV-NL63-neutralising antibody titres in plasma from 100 adult blood donors. An NT_50_ of 10 (1:10 dilution of plasma) was the limit of sensitivity for the assay.

Neutralising antibodies against HCoV-NL63 were detected within the sensitivity range of our assay in 71 (71%) of the plasma samples. The highest neutralising titre (GMT) detected was 63 with a median titre of 14. At the lowest dilution in our assay tested (1:10), neutralising antibody was undetectable or at the limit of detection in 32% of the samples (Figure 1). The largest proportion (42%) had titres between 10 and 19.9. Only 8% had titres of 30 or higher.

**Figure 2:**
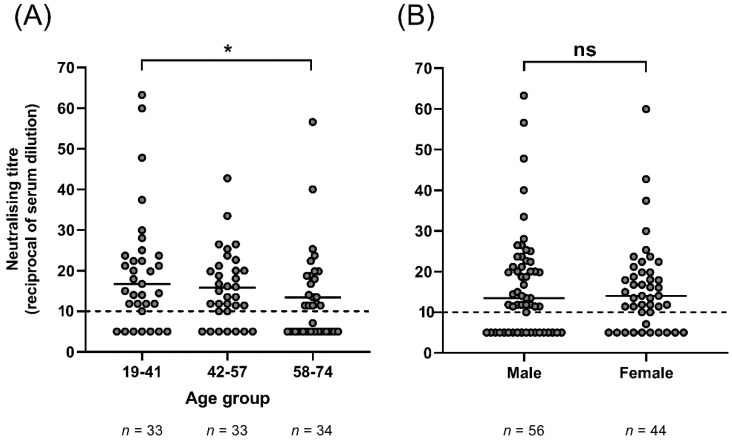
Age group (**A**) and sex (**B**) comparison of GMT. (**A**) 58–74 year olds demonstrated significantly lower median HCoV-NL63-neutralising antibody titre compared to 19–41 year olds (* *p* = 0.0485, Kruskal–Wallis test). An NT_50_ of 10 (1:10 dilution of plasma) was the limit of sensitivity for the assay. (**B**) There was no significant difference between median neutralising titres for males and females. HCoV-NL63 neutralization was detected in 38 males (68% of total males) and 33 females (75% of total females) (*p* = 0.98; Mann–Whitney *U* test).

The incidence of HCoV-NL63 infection decreases with age [16], and so we investigated if there was any correlation between age and neutralising antibody titre. Non-parametric data were log_10_ transformed and analysed with a linear regression. Goodness of fit R^2^ was 0.0428 with a slope that deviated significantly from zero (*p* = 0.0388; Figure S1), suggesting that neutralising antibody titre decreased with increasing age. This age-related reduction in GMT was evident when three evenly distributed age groups (19–41, 42–57, 58–74 years old) were compared, with significantly reduced neutralising antibody titre identified in the 58–74-year-old age group compared to the 19–41-year-old age group (*p* = 0.0485; Kruskal–Wallis test; Figure 2A). This suggests either a potential waning of the immune response to infection and/or reduced exposure to HCoV-NL63 with age.

In some studies, it has been reported that males are more susceptible to infection with HCoV-NL63 [16], implying that they may be more likely to be seropositive, or have higher titres of neutralising antibodies than females. In our study, 38/56 males (68%) had detectable neutralising antibodies to HCoV-NL63 compared to 33/44 females (75%). This difference was not statistically significant (*p* = 0.51, Fisher’s exact test). In addition, there was no difference in the median GMT between males (13.5) and females (14) (*p* = 0.98; Mann–Whitney *U* test, Figure 2B).

## 4. Discussion

In the context of the COVID-19 pandemic, there are a growing number of studies on the seroprevalence of antibodies to HCoV-NL63 [8,17,18]. Most have utilised serological assays that test for recognition of viral peptides that may not reflect functional antibody (neutralising) activity, making comparisons to our study difficult. Recent studies have quantified virus neutralising antibody in convalescent sera from SARS-CoV-2-infected individuals, and from SARS-CoV-1 patients infected in previous years. One such study used sera from 293 individuals infected with SARS-CoV-2 and a plaque reducing neutralisation assay (PRNT_50_) to quantify longitudinal neutralising antibody responses from time of infection to 7 months post-infection. The range of neutralising antibody titres detected among individuals with asymptomatic SARS-CoV-2 infection when tested from 3–7 months post-infection [19] was similar to those we observed in this study for HCoV-NL63 (from undetectable to 60). In contrast, patients with symptomatic COVID-19 had much higher neutralising antibody titres at a similar time after infection. Similarly, neutralising antibody titres to SARS-CoV-1 in sera from convalescent symptomatic patients did not fall below the limit of detection (1:10 dilution of sera) until 24 months post-infection, with a mean titre of 32 and neutralising antibody undetectable in 16% of patient sera at 36 months post-infection [20]. In addition, the previously mentioned study involving healthy adult males in Amsterdam found that antibodies to the nucleocapsid (N) protein of all seasonal coronaviruses were reduced by 50% within 6 months post-infection and returned to pre-infection baseline within 3 years [7]. The low GMT in blood samples collected in late winter, when a peak in HCoV-NL63 infection can be expected [21], suggests that the HCoV-NL63 neutralising antibody titres among healthy adults in Australia reflect a lack of recent infection.

When considering HCoV-NL63 as a model coronavirus for human challenge studies, a fundamental concern is whether the frequency of detectable neutralising antibody in a population and the titre of neutralising antibody may render study subjects refractory to experimental challenge. Historic human challenge experiments using another seasonal coronavirus, HCoV-229E, may provide some insight [22]. In these studies, the success of challenge infection and disease development was reduced as pre-challenge titres of neutralising antibody increase. However, low titres of neutralising antibody did not preclude the establishment of infection or the development of symptomatic disease [22]. Bradburne et al. found that only 25% of volunteers with high neutralising antibody titres could be infected with HCoV-229E, compared to 78% of volunteers with low titres [23]. Callow et al. also found that volunteers who were not successfully infected, or had asymptomatic infections, had higher pre-challenge neutralising antibody titres than volunteers who developed symptomatic infections [24,25]. However, 10 of 15 adult volunteers became infected, with eight volunteers developing symptomatic infection, suggesting that pre-existing neutralising antibody titres are not sufficient to protect from infection in the majority of healthy adults. When volunteers were re-challenged with HCoV-229E 12 months after their first infection, six of nine volunteers who were infected successfully the first time were re-infected, most likely due to a decline in neutralising antibody levels over 12 months to near baseline pre-infection levels. However, symptoms and virus shedding were reduced in the second infection compared to the first [25]. Unfortunately, as neutralising antibody titres were not measured using a neutralisation assay in these HCoV-229E challenge studies, results cannot be directly compared to our results. However, these findings suggest that protection from reinfection, or experimental challenge, with the same strain of HCoV does not last more than 12 months in most adults.

Both influenza A viruses and rhinoviruses have previously been used in human challenge studies. In studies with influenza, pre-existing immunity, including HA-specific antibody titres, limited viral shedding in healthy adult volunteers, although mild to moderate influenza disease was still induced [26]. Rhinovirus challenge models have been used primarily to investigate exacerbations of asthma and chronic obstructive pulmonary disease. Subjects are routinely screened for existing neutralising antibodies to the challenge strain prior to challenge and excluded if neutralising antibodies are detected in serum [27,28]. This exclusion criterion is viable for rhinovirus challenge studies due to the sero-diversity of circulating rhinoviruses. Exclusion based on the detection of even low titres of neutralising antibody to circulation season influenza or HCoVs would be more difficult in a human challenge model for these viruses.

## 5. Conclusions

Overall, our findings suggest that the background prevalence and titre of neutralising antibody to HCoV-NL63 does not preclude recruitment from the Australian population of susceptible subjects into human challenge studies in Australia. Although neutralising antibodies were detectable in 71% of donors, the median titres were low, and based on data from successful HCoV-229E challenge studies, would likely enable successful infection in most volunteers.

## Data Availability

The data set analysed is available from the authors upon reasonable request.

## Supplementary Materials

The following are available online at https://www.mdpi.com/article/10.3390/v13081618/s1, Figure S1: Log transformed GMT for 100 donors relative to age.
