# Supplementary material for: Prevalence of Neutralising Antibodies to HCoV-NL63 in Healthy Adults in Australia
Source: Viruses. 2021 Aug 16;13(8):1618. doi: 10.3390/v13081618 (PMC8402802; doi:10.3390/v13081618)
Supplement: Supplementary file 1 [file viruses-13-01618-s001.zip › viruses-1326891-supplementary.pdf]

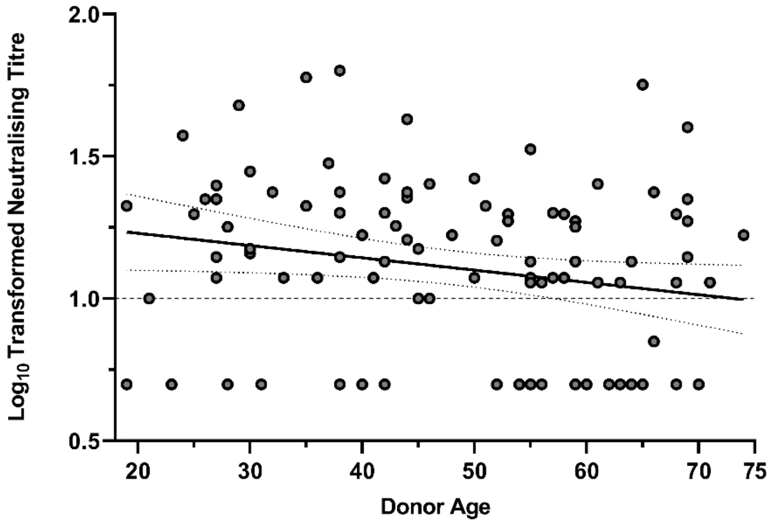

Figure S1. Log transformed GMT for 100 donors relative to age. Simple linear regression R^2 = 0.0428 with significant deviation from zero (*P* = 0.0388). Dotted lines indicate 95% Confidence Intervals, dashed line indicates lower limit of sensitivity at 1.0 $\log_{10}$.
